# Downregulation of oar-miR-125b Drives Blood–Brain Barrier Breakdown Through the TNFSF4–NF-κB Inflammatory Axis in Enterococcus Faecalis Meningitis

**DOI:** 10.3390/microorganisms13122644

**Published:** 2025-11-21

**Authors:** Longling Jiao, You Wu, Borui Qi, Pengfei Zhao, Ming Zhou, Runze Zhang, Yongjian Li, Jingjing Ren, Shuzhu Cao, Yayin Qi

**Affiliations:** College of Animal Science & Technology, Shihezi University, Shihezi 832003, China; 13364947693@163.com (L.J.); wuyou1180@163.com (Y.W.); 18709646925@163.com (B.Q.);

**Keywords:** bacterial meningitis, oar-miR-125b, TNFSF4, inflammation, blood–brain barrier

## Abstract

Bacterial meningitis involves complex molecular networks, including microRNA-mediated regulation of inflammatory responses; however, the specific role of Ovis aries microRNA-125b (oar-miR-125b) in this process remains poorly understood. In this study, using a lamb model of Enterococcus faecalis-induced meningitis, we observed significant downregulation of oar-miR-125b, which inversely correlated with its newly identified target, Tumor Necrosis Factor Superfamily Member 4 (TNFSF4). Dual-luciferase reporter assays confirmed that oar-miR-125b directly binds to the 3′ Untranslated Region (3′UTR) of TNFSF4 but not to the 3′UTRs of Kelch Like Family Member 31 (KLHL31) or NF-κB Inhibitor Interacting Ras Like 2 (NKIRAS2). Mechanistically, decreased oar-miR-125b expression relieves its repression of TNFSF4, leading to NF-κB pathway activation and blood–brain barrier disruption. Collectively, our results demonstrate that oar-miR-125b serves as a key anti-inflammatory regulator in bacterial meningitis by targeting TNFSF4 and constraining NF-κB signaling, highlighting its potential as a therapeutic target for attenuating neuroinflammation in meningitis.

## 1. Introduction

The blood–brain barrier (BBB) is a key structural component of the neurovascular unit and strictly regulates molecular exchange between the circulation and the brain parenchyma. Its disruption is a hallmark of numerous central nervous system (CNS) pathologies, underscoring the importance of understanding its compromise during bacterial meningitis. Bacterial meningitis is an acute suppurative infection of the CNS caused by direct bacterial invasion, marked by sudden onset, rapid progression, and high mortality rates [1]. Globally, it poses a substantial health burden with pronounced geographical disparities: annual incidence ranges from approximately 0.9 per 100,000 individuals in high-income countries to as high as 80 per 100,000 in resource-limited regions [2]. In areas with constrained healthcare infrastructure, mortality rates can reach 54%, and nearly one-quarter of survivors develop chronic neurological sequelae, including sensorineural hearing loss, cognitive deficits, and motor impairments [3]. The incidence of bacterial meningitis demonstrates substantial geographical and age-dependent heterogeneity, with newborns bearing a disproportionately high burden of disease and mortality. This burden is driven largely by Group B Streptococcus (GBS), which is the dominant pathogen in infants under two months of age, accounting for 46% of cases in this group compared to fewer than 4% in those aged two months or older [4]. As a serious condition often associated with invasive infections such as pneumonia, bacteremia, and sepsis, bacterial meningitis remains a critical priority for global public health initiatives. Epidemiological studies indicate that Enterococcus faecalis, while accounting for only 0.3–4% of bacterial meningitis cases, is of particular concern due to its pathogenic versatility [5]. Although commonly used as a probiotic in animal models to improve digestive function and intestinal health [6], Enterococcus faecalis is a commensal gut bacterium in humans that can translocate across the BBB following events such as traumatic brain injury or neurosurgery, rapidly leading development of purulent meningitis. Beyond the CNS, Enterococcus faecalis is the third most common cause of infective endocarditis worldwide, accounting for 10–15% of cases, with this strain accounting for nearly 90% of enterococcal-related incidents [7]. Enterococcus faecalis is also a major pathogen in complicated urinary tract and intra-abdominal infections [8]. In China, hospital-acquired enterococcal infections are increasingly prevalent. Pathogenicity studies reveal that specific strains can induce distinct clinical syndromes in different animal hosts, including swine septicemia, bovine mastitis, and lamb meningitis. Treatment of Enterococcus faecalis meningitis is particularly challenging, owing to its intrinsic resistance to β-lactams and cephalosporins, coupled with its capacity to acquire resistance genes—via plasmid transfer—against macrolides, glycopeptides, and tetracyclines, which severely limiting therapeutic options [9]. Given these challenges, elucidating the molecular mechanisms governing Enterococcus faecalis infection, particularly its ability to disrupt the BBB, is of critical importance.

The BBB represents a highly selective interface between the CNS and the peripheral circulation. Its core physical barrier is primarily established by tight junctions between brain microvascular endothelial cells. These junctions consist of transmembrane proteins—such as Claudin-5, Claudin-3, and Occludin—which are anchored to the actin cytoskeleton by cytoplasmic scaffolding proteins including ZO-1, ZO-2, and ZO-3 [10]. Together with vascular endothelial cadherin and junctional adhesion molecules, these components assemble into a sealed structure that is nearly impermeability to ions and macromolecules. Homeostasis of the BBB is maintained not only by these endothelial complexes but also through sophisticated transcellular crosstalk that involves soluble signals secreted by pericytes, astrocytic end-feet, microglia, oligodendrocytes, and neurons [11]. In addition to its physical barrier function, the BBB mediates essential selective transport functions. Whereas small hydrophobic molecules may diffuse passively, essential nutrients such as glucose and amino acids require specific carrier-mediated transporters. Moreover, many potential neurotherapeutic agents are effectively excluded by ATP-binding cassette (ABC) efflux pumps expressed at the endothelial membrane. Under pathological conditions such as acute ischemic stroke, the dysregulated upregulation of aquaporins exacerbates vasogenic edema, while simultaneous ABC transporter activity expels neuroprotective agents from the brain parenchyma—together creating a detrimental “dual barrier” effect [12]. In a range of neurological disorders—including stroke, multiple sclerosis, glioblastoma, and traumatic brain injury—the structural integrity of the BBB is compromised [13,14]. This disruption, compounded by aberrant transport mechanisms, permits the entry of neurotoxic substances and the expulsion of therapeutic molecules, thereby amplifying neural injury and impeding recovery. Therefore, future intervention must simultaneously aim to restore the barrier and normalize transport function to achieve precision therapeutics for CNS diseases.

MicroRNAs (miRNAs) are single-stranded, non-coding RNAs, approximately 19–22 nucleotides in length, that post-transcriptionally regulate gene expression. Following their maturation, they guide the RNA-induced silencing complex (RISC) to complementary sequences in the 3′UTRs of target mRNAs [15]. This interaction typically leads to either mRNA degradation or translational repression, effectively silencing gene expression. Among the diverse families of miRNA, miR-125b has attracted considerable interest due to its high evolutionary conservation and context-dependent regulatory roles. In cancer biology, miR-125b exhibits dual functionality, acting as either a tumor suppressor or an oncogene depending on the cellular context. For instance, it inhibits cell proliferation by targeting MKNK2 [16], and suppresses invasion and metastasis in gastric cancer by directly repressing STAT3 [17]. Conversely, miR-125b promotes metastatic progression in colorectal cancer by downregulating CFTR and CGN. This mechanism is supported by the inverse correlation between miR-125b levels and CFTR/CGN expression in both primary tumors and distant metastases, highlighting its potential as a biomarker for disease aggressiveness [18]. Furthermore, in HPV-driven carcinogenesis, the viral oncoprotein E6 facilitates immune evasion by suppressing miR-125b, which normally targets TAZ. This attenuation of Hippo pathway signaling accelerates cervical cancer progression [19]. In the realm of inflammatory regulation, miR-125b acts as a rapid-response modulator to extracellular stimuli such as TLR4 activation. By directly targeting key negative regulators of the NF-κB pathway—including TNFAIP3 (A20) [20] and NKIRAS2 [21]—miR-125b derepresses the pathway’s intrinsic inhibitory feedback, leading to sustained NF-κB activation and elevated production of proinflammatory cytokines such as TNF-α. Conversely, in cardiovascular pathophysiology, miR-125b-5p serves a protective role by targeting TNFSF4, thereby attenuating the TLR4/NF-κB signaling axis, reducing oxidative stress, and mitigating ox-LDL-induced endothelial injury [22]. In summary, miR-125b functions as a versatile regulatory node that fine-tunes tumor progression, inflammatory intensity, and cardiovascular homeostasis in a context-specific manner. Its functional pleiotropy and conservation highlight its strong potential as both a diagnostic biomarker and a therapeutic target in a range of human diseases.

The OX40 ligand (OX40L), a transmembrane glycoprotein encoded by TNFSF4 and a member of the TNF superfamily, serves as a critical co-stimulatory molecule on antigen-presenting cells (APCs) to deliver a potent secondary activation signal to T cells via engagement with its receptor OX40 (TNFRSF4) [23]. This ligation triggers intracellular signaling primarily through the OX40-TRAF-NF-κB/PI3K axis, ultimately promoting T cell clonal expansion, survival, effector differentiation, and the formation of memory populations, thereby substantially amplifying adaptive immune responses [24]. Given the potency of this pathway, its precise immunological control is essential. Dysregulation or overactivation can breach immune tolerance, leading to the expansion of autoreactive T cells that drive the pathogenesis of autoimmune diseases such as systemic lupus erythematosus (SLE) [25] and rheumatoid arthritis (RA) [26]. Furthermore, in chronic inflammatory conditions like atherosclerosis, sustained OX40L signaling exacerbates plaque inflammation and progression [27]. In tumor immunology, the TNFSF4/OX40L axis displays dual functions. In some scenarios, regulatory T cells (Tregs) within the tumor microenvironment can co-opt this axis to promote immunosuppression and facilitate immune evasion [28]. Conversely, strategic activation of this pathway with specific antibodies can reverse T cell exhaustion and potently enhance the cytotoxic capacity of CD8+ T cells, reinvigorating anti-tumor immunity [29]. This functional duality has prompted the development of two distinct therapeutic strategies: using antagonistic antibodies to block the pathway for treating autoimmunity and transplant rejection and applying agonistic antibodies to amplify signaling for cancer immunotherapy. Thus, the TNFSF4/OX40L axis has emerged as a pivotal and tunable therapeutic target spanning a broad spectrum of human diseases, including autoimmune disorders, chronic inflammation, and malignancies.

## 2. Materials and Methods

### 2.1. Cell Culture

Primary ovine brain microvascular endothelial cells (OBMECs) (Cellverse, Shanghai, China) were cultured in complete endothelial cell medium (Cellverse, Shanghai, China). For oar-miR-125b target validation via dual-luciferase assays, HEK293T cells (ATCC CRL-3216) from the Key Laboratory of Preventive Veterinary Medicine (Shihezi University), were cultured in high-glucose DMEM (Gibco, Grand Island, NY, USA) supplemented with 10% fetal bovine serum(Excell, Shanghai, China). Both cell lines were incubated at 37 °C in a 5% CO_2_ atmosphere.

### 2.2. Enterococcus Faecalis-Infected OBMECs

Select clinical isolates of Enterococcus faecalis causing meningitis from the laboratory collection and culture them in BHI broth until the logarithmic growth phase. Passage primary OBMECs to the third generation and seed them at 1.0 × 10^5^ cells per well in a 6-well plate. When cell confluence reaches 80–90%, establish an infection group and a control group. The control group received endothelial cell complete medium supplemented with an equal volume of PBS instead of bacterial solution. The infected group received log-phase Enterococcus faecalis at a 10 MOI to infect the cells. After 2 h, the medium was replaced with gentamicin-containing medium to kill extracellular bacteria. After 24 h, RNA was extracted and analyzed by qPCR to assess changes in the expression levels of oar-miR-125b, TNFSF4, and inflammatory factors.

### 2.3. Cell Transfection

oar-miR-125b mimics, inhibitors, and their corresponding negative controls were synthesized by Genepharma (Shanghai, China). Primary sheep brain microvascular endothelial cells were seeded into 6-well plates (1 × 10^5^ cells/well) and transfected with oligonucleotides (60 nM) using Lipofectamine 3000 (Invitrogen, Carlsbad, CA, USA) for 24 h.

### 2.4. Dual-Luciferase Reporter Assay

The predicted oar-miR-125b binding site within the TNFSF4 3′-UTR and its mutated counterpart were synthesized and cloned into the GP-mirGLO vector (GenePharma, Shanghai, China) to generate wild-type (WT-TNFSF4) and mutant (mut-TNFSF4) reporter constructs. For the luciferase assay, OBMECs were co-transfected with 50 ng of the respective reporter vector and either 20 nM oar-miR-125b mimic or negative control (miR-NC). After 48 h, luciferase activities were measured using a dual-luciferase reporter assay system (GenePharma, Shanghai, China) according to the manufacturer’s instructions.

### 2.5. Quantitative Real-Time PCR Analysis

RNA samples were obtained using the EasyPure Fast Cell RNA Kit (TransGen Biotech, Beijing, China). For miRNA, complementary DNA (cDNA) was synthesized using the miRNA 1st Strand cDNA Synthesis Kit (Vazyme Biotech, Nanjing, China). For mRNA, cDNA was synthesized using the TransScript Uni All-in-One First-Strand cDNA Synthesis SuperMix for qPCR (TransGen Biotech, Beijing, China). qPCR for miRNA and mRNA levels was performed using ChamQ Universal SYBR qPCR Master Mix (Vazyme Biotech, Nanjing, China). Primers listed in Table 1 were synthesized by Xinjiang Youkang Biotechnology (Urumqi, China). Gene expression levels were calculated using the 2^−ΔΔCt^ method. The primer sequences are provided in Table 1.

### 2.6. Western Blot Analysis

Total protein from OBMECs was extracted using RIPA lysis buffer and quantified with a BCA kit (Biosharp, Hefei, China). Proteins (30 µg) were separated by SDS-PAGE, transferred to a PVDF membrane (Millipore, Burlington, MA, USA), and blocked with 5% non-fat milk. The membrane was then incubated overnight at 4 °C with primary antibodies against ZO-1, Claudin-5, Occludin, NF-κB p65, Phospho-NF-κB p65 (all from Proteintech, Wuhan, China), and β-actin (Servicebio, Wuhan, China). Following incubation with an HRP-conjugated secondary antibody (Proteintech, Wuhan, China), protein bands were visualized using an ECL substrate (Thermo Fisher, Waltham, MA, USA) and a chemiluminescence imager (Servicebio, Wuhan, China). Band intensities were quantified with ImageJ 1.53q (National Institutes of Health, Bethesda, MD, USA).

### 2.7. Immunofluorescence

Immunofluorescence was performed to examine the expression and localization of tight junction proteins in OBMECs. Upon reaching 100% confluence, cells were fixed with 4% paraformaldehyde for 30 min and permeabilized with 0.3% Triton X-100 for 5 min at room temperature. After blocking with 5% BSA for 2 h, the cells were incubated overnight at 4 °C with primary antibodies against ZO-1, Occludin, and Claudin-5 (all from Proteintech, Wuhan, China). Following three washes with PBST, the cells were incubated for 1 h with a Multi-rAb CoraLite^®^ Plus 488-Goat Anti-Rabbit Recombinant Secondary Antibody (H + L) (Proteintech, Wuhan, China). Nuclei were counterstained with 0.5 μg/mL DAPI (Beyotime, Shanghai, China) for 8 min. Fluorescence images were acquired using a fluorescence microscope (SOPTOP, Ningbo, China) and merged using ImageJ 1.53q (National Institutes of Health, Bethesda, MD, USA).

### 2.8. Statistical Analysis

After verifying assumptions of normality and homogeneity of variances, pairwise comparisons were analyzed using two-tailed unpaired *t*-tests. For multiple group comparisons, use one-way or two-way ANOVA. Data are presented as mean ± SEM. Statistical analyses were conducted using GraphPad Prism 8.0 (GraphPad Software, San Diego, CA, USA). Significance levels were defined as * *p* < 0.05, ** *p* < 0.01, and *** *p* < 0.001; “ns” indicates non-significance.

## 3. Results

Following the initial observation of marked oar-miR-125b downregulation in brain tissue from our lamb bacterial meningitis model [30], a subsequent investigation was conducted to elucidate the underlying mechanism. Through a comprehensive mechanistic exploration employing in vitro BBB models and molecular biology approaches, this study uncovered how the oar-miR-125b/TNFSF4/NF-κB axis orchestrates inflammatory cascades and disrupts blood–brain barrier integrity.

### 3.1. Potential Negative Regulation of the Proinflammatory Factor TNFSF4 by oar-miR-125b

**Figure 1:**
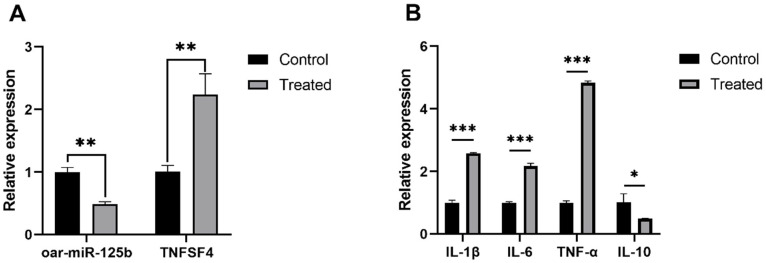
Enterococcus faecalis infection downregulates oar-miR-125b and upregulates proinflammatory factors in OBMECs. (**A**) Expression levels of oar-miR-125b and TNFSF4 were detected by qPCR in OBMECs after Enterococcus faecalis infection. (**B**) qPCR analysis of IL-1β, IL-6, TNF-α, and IL-10 expression levels in OBMECs post-Enterococcus faecalis infection. * *p* < 0.05, ** *p* < 0.01, *** *p* < 0.001 (compared with the control group). Control represents untreated cells; Treated represents cells infected with Enterococcus faecalis.

To investigate the expression regulation relationship between miR-125b and its potential target gene TNFSF4 in Enterococcus faecalis infection, this study employed Enterococcus faecalis to treat primary OBMECs to establish an in vitro Enterococcus faecalis infection model. qPCR analysis revealed that compared to the control group, mRNA expression of IL-1β, IL-6, TNF-α, and TNFSF4 was significantly upregulated in the treated group, while IL-10 and miR-125b expression levels were significantly suppressed (Figure 1A,B). The significant negative correlation in their expression patterns aligns with the typical characteristic of microRNA negatively regulating its target genes. Given that TNFSF4 is a known proinflammatory factor, these results suggest miR-125b may participate in negative feedback regulation of inflammation by targeting TNFSF4. Combining this with existing literature confirming that human miR-125b directly targets TNFSF4 [15], we speculate that TNFSF4 is also a direct target of miR-125b in sheep.

### 3.2. TNFSF4 Has Been Identified as a Direct Target of oar-miR-125b

**Figure 2:**
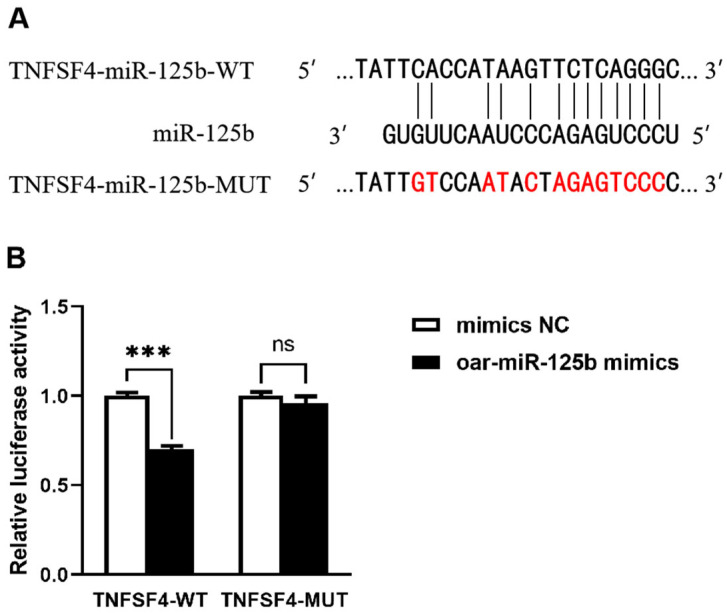
Targeting relationship between TNFSF4 and oar-miR-125b. (**A**) The target bindings sites between oar-miR-125b and the 3′-UTR of TNFSF4 gene. (**B**) oar-miR-125b overexpression inhibited the luciferase activity of HEK-293 cells transfected with wild-type sequence of TNFSF4, while did not affect those with mutant sequence of TNFSF4, *** *p* < 0.001 compared with miR-NC. Non-significant differences are indicated by ns. The red-marked bases are the mutant bases targeting the binding site in the TNFSF4 mutant (MUT).

To validate TNFSF4 as a hypothesized direct target of oar-miR-125b, we initially employed bioinformatics analysis, which identified a specific complementary sequence for oar-miR-125b within the 3′UTR of the TNFSF4 gene (Figure 2A). We subsequently validated this interaction using a dual-luciferase reporter assay in 293T cells. The results showed that oar-miR-125b overexpression significantly suppressed the luciferase activity of a reporter construct containing the wild-type (WT) TNFSF4 3′UTR but had no significant effect on a construct bearing a mutated (MUT) binding site (Figure 2B). These findings confirm a direct and specific binding interaction between oar-miR-125b and the TNFSF4 3′UTR.

**Figure 3:**
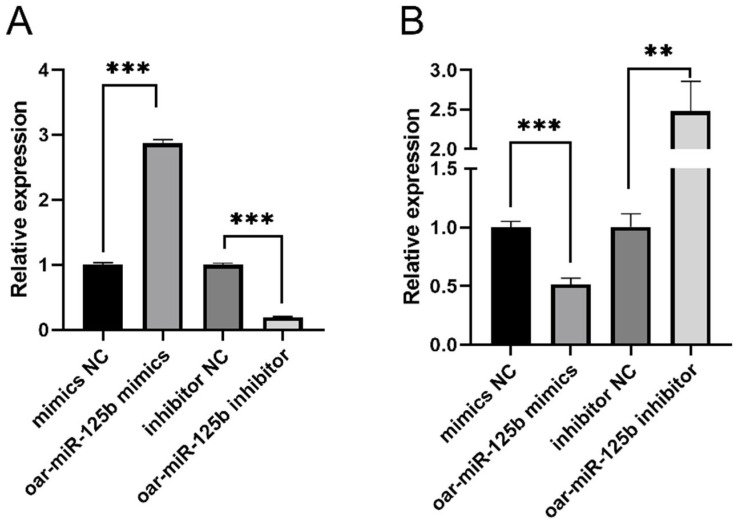
Oar-miR-125b suppresses TNFSF4 expression in OBMECs. (**A**) The transfection efficiency of oar-miR-125b mimic or inhibitor in OBMECs as measured by qPCR. (**B**) qPCR analysis of TNFSF4 in OBMECs transfected with oar-miR-125b mimic or inhibitor. ** *p* < 0.01, *** *p* < 0.001 compared with miR-NC.

To functionally validate the targeting of TNFSF4 by oar-miR-125b in OBMECs, we performed gain- and loss-of-function experiments by transfecting cells with oar-miR-125b mimics, inhibitors, or their respective negative controls (NC). Transfection efficiency was confirmed by qPCR at 24 h post-transfection (Figure 3A). Subsequent analysis of TNFSF4 mRNA levels showed that oar-miR-125b overexpression led to a highly significant downregulation (*p* < 0.0001), whereas its inhibition resulted in a significant upregulation (*p* < 0.001), compared to the NC group (Figure 3B). These complementary results provide direct evidence that oar-miR-125b acts as a negative regulator of TNFSF4 expression in OBMECs.

In summary, this study collectively confirmed that TNFSF4 is a direct target of oar-miR-125b through bioinformatics prediction, dual luciferase reporter assays, and functional validation at the cellular level. Given the established role of TNFSF4 as a key upstream regulator of the NF-κB signaling pathway, these findings suggest that oar-miR-125b may negatively regulate the NF-κB pathway and downstream inflammatory responses by specifically inhibiting TNFSF4.

### 3.3. KLHL31 and NKIRAS2 Were Confirmed Not to Be Direct Targets of Sheep oar-miR-125b

**Figure 4:**
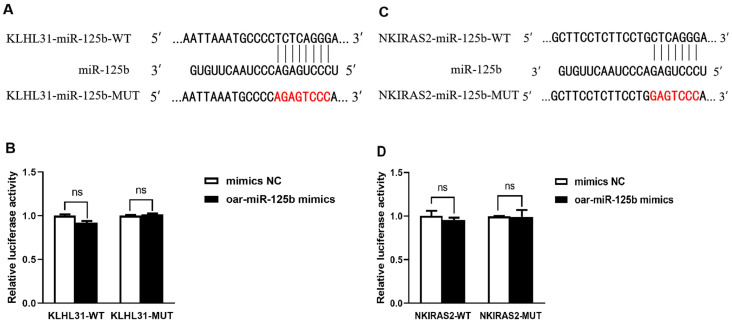
KLHL31 and NKIRAS2 do not have a targeting relationship with oar-miR-125b. (**A**,**C**) The target bindings sites between oar-miR-125b and the 3′-UTR of KLHL31 and NKIRAS2 gene. (**B**,**D**) oar-miR-125b overexpression inhibited the luciferase activity of HEK-293 cells transfected with wild-type sequence of KLHL31 and NKIRAS2, while did not affect those with mutant sequence of TNFSF4. Non-significant differences are indicated by ns. The red-labeled bases represent the mutant nucleotides at the target binding sites in KLHL31 and NKIRAS2 mutants (MUT).

To systematically identify potential targets of oar-miR-125b, we performed bioinformatics screening, which indicated KLHL31 as a candidate gene harboring a putative binding site for oar-miR-125b in its 3′UTR (Figure 4A). To experimentally validate this prediction, we conducted a dual-luciferase reporter assay in 293T cells. The results showed that oar-miR-125b overexpression did not produce statistically significant suppression of luciferase activity for either the wild-type (WT) or mutant (MUT) KLHL31 3′UTR reporter constructs (Figure 4B). Consequently, these findings rule out a direct regulatory relationship between oar-miR-125b and the KLHL31 3′UTR under the tested conditions.

While miR-125b has been established as a direct regulator of NKIRAS2 in cattle [21], its functional conservation in sheep remained unclear. Bioinformatics analysis initially predicted a conserved binding site in the ovine NKIRAS2 3′UTR (Figure 4C). However, sequence alignment revealed only 70% homology in the 3′UTR region between sheep and cattle, despite high conservation of the mature miR-125b sequence itself (Figure S1). This discrepancy suggested potential species-specificity in the targeting relationship. Functional validation using a dual-luciferase reporter assay confirmed this hypothesis: overexpression of oar-oar-miR-125b did not significantly suppress the activity of the wild-type ovine NKIRAS2 3′UTR reporter (*p* > 0.05; Figure 4D). This result indicates that NKIRAS2 is not a direct target of oar-miR-125b in sheep. We postulate that nucleotide substitutions within the seed-matching region of the sheep NKIRAS2 3′UTR may preclude effective binding to oar-miR-125b, thereby abrogating the regulatory interaction observed in cattle.

### 3.4. oar-miR-125b Negatively Regulates the NF-κB Pathway and Its Mediated Inflammatory Response by Targeting and Inhibiting TNFSF4

**Figure 5:**
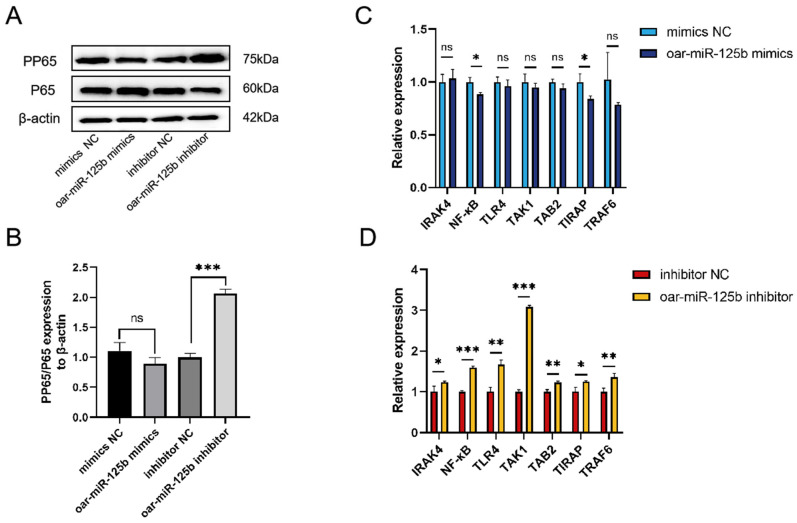
Oar-miR-125b regulates NF-κB p65 phosphorylation and signaling. (**A**,**B**) Western blot analysis of PP65 and P65 expression in OBMECs following transfection with oar-miR-125b mimic or inhibitor. Western blot images were analyzed using the Image J software, and densitometry quantification of Western blots were normalized to β-actin. (**C**,**D**) Following transfection of oar-miR-125b mimics or inhibitors into OBMECs, real-time quantitative PCR was employed to detect the expression of mRNA molecules associated with the NF-κB signaling pathway. * *p* < 0.05, ** *p* < 0.01, *** *p* < 0.001 compared with miR-NC. Non-significant differences are indicated by ns.

To elucidate the regulatory function of oar-miR-125b in the inflammatory response of ovine brain microvascular endothelial cells (OBMECs), we modulated its expression using mimic and inhibitor transfection. Subsequent qPCR and Western blot analyses revealed that inhibition of oar-miR-125b significantly enhanced NF-κB pathway activation, as evidenced by increased phosphorylation of the NF-κB protein (Figure 5A,B) and upregulation of key pathway component transcripts (Figure 5D). In contrast, oar-miR-125b overexpression did not produce a significant suppressive effect under the tested conditions (Figure 5A–C). Collectively, these results demonstrate that oar-miR-125b functions as a tonic inhibitor of the NF-κB signaling pathway and its downstream inflammatory response by targeting TNFSF4. The loss of oar-miR-125b expression consequently relieves this repression, leading to heightened inflammatory activation.

### 3.5. oar-miR-125b Is a Positive Regulator of Blood–Brain Barrier Integrity

**Figure 6:**
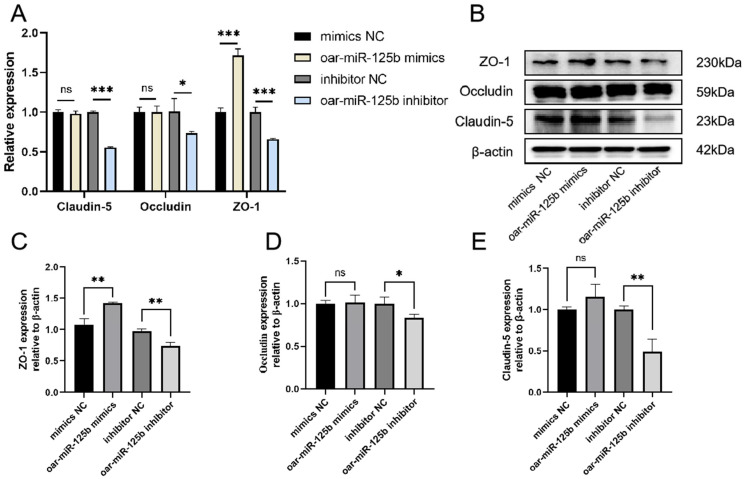
Oar-miR-125b regulates tight junction protein expression. (**A**) Following transfection of oar-miR-125b mimics or inhibitors into OBMECs, the expression of tight junction-associated mRNA molecules was detected using real-time quantitative PCR. (**B**–**E**) Western blot analysis of changes in Core-binding protein in OBMECs following transfection with oar-miR-125b mimic or inhibitor. Western blot images were analyzed using the Image J software, and densitometry quantification of Western blots were normalized to β-actin. * *p* < 0.05, ** *p* < 0.01 *** *p* < 0.001, compared with miR-NC. Non-significant differences are indicated by ns.

To directly assess the functional contribution of oar-miR-125b to Blood–Brain Barrier integrity, we modulated its expression in OBMECs using specific inhibitors and mimics. Analysis of core tight junction proteins by Western blot, qPCR and IF staining revealed that oar-miR-125b knockdown significantly reduced both the mRNA and protein levels of ZO-1, claudin-5, and occludin, which was further corroborated by a pronounced decrease in their fluorescent signal intensity and continuity at cell-cell junctions. Conversely, oar-miR-125b overexpression markedly upregulated their expression (Figure 6A–E and Figure S2). These complementary gain- and loss-of-function data establish oar-miR-125b as a critical positive regulator essential for maintaining tight junction integrity in OBMECs.

## 4. Discussion

As a pivotal regulator molecule within the central nervous system (CNS), miR-125b contributes to the blood–brain barrier (BBB) homeostasis through targeting multiple signaling pathways across pathological conditions. Experimental data indicate that miR-125b expression is significantly downregulated in a lamb Enterococcus faecalis meningitis model [30], in stark contrast to reports of increased expression in viral encephalitis models [31], suggesting distinct pathogen-specific regulation of oar-miR-125b expression. In this study, we identified TNFSF4 as a direct target of oar-miR-125b through dual-luciferase reporter assays and qPCR validation. Furthermore, we revealed species-specificity regulatory patterns, as the previously reported targeting of NKIRAS2 by miR-125b in bovine models [21] was not conserved in our ovine system. Importantly, our findings demonstrate that oar-miR-125b downregulation and subsequent TNFSF4/NF-κB pathway activation represent key early events in BBB disruption. This discovery suggests that monitoring oar-miR-125b levels in blood or CSF, along with TNFSF4 concentrations, could serve as a novel, minimally invasive diagnostic approach for identifying patients at high risk of CNS complications caused by Enterococcus faecalis infection.

We further uncovered a self-reinforcing feedback mechanism within the pathway: sustained NF-κB activation may further maintain low miR-125b expression through epigenetic regulation, creating a cascading inflammatory cycle. This finding integrates evidence from diverse CNS disease models into a unified framework positioning miR-125b as a central coordinator of BBB protection. Its multifunctional role encompasses enhancing endothelial acid tolerance through acid-sensing ion channel 1 (ASIC1) [32] suppression in cerebral ischemia, maintaining vascular stability by regulating the RTEF-1/VEGF axis in high-altitude cerebral edema [33], and mitigating barrier structural damage by influencing factors like MMP-15 in traumatic brain injury [34]. Collectively, these insights establish miR-125b as a core regulator within a coordinated defense network spanning metabolic adaptation, functional regulation, and structural stabilization of the neurovascular unit, highlighting its therapeutic potential for cerebrovascular and neuroinflammatory disorders.

Beyond its well-characterized roles in other pathological contexts, miR-125b has emerged as a context-dependent regulator of inflammatory responses. Previous studies have shown that miR-125b suppresses NF-κB activation through direct targeting of TRAF6 in sepsis [35] and promotes inflammation via A20 inhibition in rheumatoid arthritis [20]. In this study, miR-125b attenuates neuroinflammation by directly targeting TNFSF4 and inhibiting NF-κB signaling. Consistent with recent observations that miR-125b serves as a context-dependent rheostat whose pro- or anti-inflammatory output is determined by cell-type-specific target repertoires and the pathological microenvironment [36,37], our data further underscore its dichotomous yet precise regulatory role within inflammatory signaling networks. Experimentally, we established that proinflammatory mediators downregulate tight junction proteins ZO-1, Claudin-5, and Occludin, with structural disintegration of the tight junction system marking the blood–brain barrier’s transition to pathological compromise [38]. Significantly, this study is the first to link the oar-miR-125b-TNFSF4-NF-κB axis to tight junction regulation, revealing a complete signaling pathway through which inflammatory responses induce blood–brain barrier dysfunction in bacterial meningitis. These findings deepen our understanding of the mechanisms underlying blood–brain barrier disruption in bacterial meningitis from both structural and functional perspectives, providing novel theoretical foundations for developing targeted therapeutic strategies.

This study delineates the oar-miR-125b/TNFSF4/NF-κB regulatory axis, deepening our molecular comprehension of bacterial meningitis pathogenesis and underscoring its considerable translational relevance. From a diagnostic standpoint, the specific downregulation of miR-125b in peripheral blood presents a promising non-invasive biomarker capable of facilitating early and rapid discrimination between bacterial meningitis and viral CNS infections. This approach may circumvent the inherent limitations of conventional cerebrospinal fluid analysis, particularly in terms of timeliness and invasiveness. Accumulating clinical evidence confirms that while procalcitonin serves as a useful indicator of severe bacterial infection, it exhibits insufficient specificity to reliably differentiate bacterial from viral respiratory etiologies in pediatric populations [39]. Consequently, current guidelines emphasize a multidisciplinary for instance, combining procalcitonin with more recently identified markers such as gastrin, to integrate clinical assessment with complementary laboratory parameters for informing antibiotic therapy decisions [40]. The development of integrated multi-marker strategies, potentially incorporating emerging biomarkers such as miR-125b, represents a promising avenue for more accurately identifying febrile children who would benefit from antibiotic treatment. Therapeutically, targeting key nodes of this axis, such as administering exogenous oar-miR-125b mimics, restoring barrier integrity or employing TNFSF4-specific antagonists to mitigate inflammatory amplification, offers avenues for novel molecularly targeted interventions. Furthermore, the species-specific nature of the oar-miR-125b regulatory network, as identified in this study, not only clarifies functional discrepancies across experimental models but also highlights the imperative of accounting for interspecies variation in the development of targeted therapies. Together, these insights establish a conceptual foundation for advancing precision medicine in the management of bacterial meningitis.

However, this study still has several limitations. First, although the data support the role of the TNFSF4–NF-κB pathway, the underlying mechanism by which Enterococcus faecalis downregulates oar-miR-125b remains unclear; potential triggering factors, such as bacterial secretory factors or activation of host pattern recognition receptors, require systematic screening and validation. Second, while we identified a regulatory feedback loop between NF-κB and oar-miR-125b, potential underlying epigenetic mechanisms, such as promoter region methylation or histone modifications—require further experimental validation. Finally, this study validated the miR-125b–TNFSF4–NF-κB axis exclusively in cerebral microvascular endothelial cells. Its physiological relevance requires confirmation in more complex in vivo-like models. Therefore, subsequent research should validate this pathway in physiologically relevant models, such as Transwell-based blood–brain barrier co-culture systems, to assess the axis’s function and translational potential under conditions closer to in vivo conditions.

## 5. Conclusions

This study establishes the oar-miR-125b–TNFSF4–NF-κB axis as playing a critical role in bacterial meningitis pathogenesis. It not only deepens our understanding of the molecular mechanisms underlying the disease but also provides a theoretical basis for developing novel diagnostic approaches and targeted therapeutic strategies. Future research should validate these findings in more complex model systems and advance their clinical translation. Notably, a deeper understanding of miRNA regulatory networks may offer novel therapeutic approaches for various central nervous system infectious diseases, ultimately improving patient clinical outcomes.

## Figures and Tables

**Table 1:** List of primer sequences used in this study.

Target Gene	Primer Sequence (5′→3′)
oar-miR-125b	RT: GTCGTATCCAGTGCAGGGTCCGAGGTATTCGCACTGGATACGACCACAAG
TNFSF4	F: CGCGTCCCTGAGACCCTAA
	R: AGTGCAGGGTCCGAGGTATT
	F: CACGTTCCCCTTTTCCATATCT
	R: CCTCCTTTTGGGAAGTGAGGA
TLR4	F: TGTGAAGGACATGCCAGTGCTTG
	R: TGACAACCGACACGCTGATGATC
NFκB	F: ACAAGCCTGTCACAGCCAACATG
	R: TGATGGTGAAGGCTCAGGAGGTG
TAB2	F: GGAAGCAGGACTCTAACGCACAG
	R: GCCTTGAGGAACTTGAGCTGGTG
TIRAP	F: CCTCAGCAGAGCCGCCTACC
	R: GCATGACAGCGTCCTTGACTTGG
IRAK4	F: CTCAAGTGATGGCGATGACCTCTG
	R: CCATCCAAGCAAGCCAGTCTGTC
TAK1	F: TCCGCCGCTTCTTCCTCCTC
	R: GCTCCTCTTCCAACAACCTCTTCC
TRAF6	F: ACTGAGGCATCTTGAGGAGCATC
	R: TTCTGGAAGAGACGCTGGCATTG
β-actin	F: ACTGGGACGACATGGAGAAGA
	R: GCGTACAGGGACAGCACAG
IL-6	F: AGACTACTTCTGACCACTCCA
	R: TCACACTCGTCATTCTTCTCAC
TNF-α	F: GACGGGCTTTACCTCATCTAC
	R: CTTGATGGCAGAGAGGATGTT
IL1β	F: AGGAAATGAGCCGAGAAGTG
	R: ATTCTTGTCCCTGATACCCAAG
IL-10	F: TGCTGGATGACTTTAAGGG
	R: AGGGCAGAAAACGATGACA
U6	F: GCTTCGGCAGCACATATACT
	R: TTCACGAATTTGCGTGTCAT

## Data Availability

The datasets presented in this article are not readily available because the data are part of an ongoing study. Requests to access the datasets should be directed to qiyayin@shzu.edu.cn.

## Supplementary Materials

The following supporting information can be downloaded at: https://www.mdpi.com/article/10.3390/microorganisms13122644/s1, Figure S1: Homology comparison of oar-miR-125b and NKIRAS2 genes in sheep and cattle. (A) Alignment of mature sequences of oar-miR-125b in sheep and miR-125b in cattle. (B) The homology of NKIRAS2 mRNA in sheep and cattle. Figure S2: Oar-miR-125b regulates tight junction protein expression. Following transfection of OBMECs cells with oar-miR-125b mimics or inhibitors, immunofluorescence analysis was performed to detect the expression of tight junction-associated proteins.
