# Supplementary material for: Downregulation of oar-miR-125b Drives Blood–Brain Barrier Breakdown Through the TNFSF4–NF-κB Inflammatory Axis in Enterococcus Faecalis Meningitis
Source: Microorganisms. 2025 Nov 21;13(12):2644. doi: 10.3390/microorganisms13122644 (PMC12734523; doi:10.3390/microorganisms13122644)
Supplement: Supplementary file 1 [file microorganisms-13-02644-s001.zip › microorganisms-3965953-supplementary.pdf]

A bta-miR-125b 5’ -UCCCUGAGACCCUAACUUGUGA-3’ MIMAT0003539

oar-miR-125b 5’ -UCCCUGAGACCCUAACUUGUG-3’ MIMAT0014971

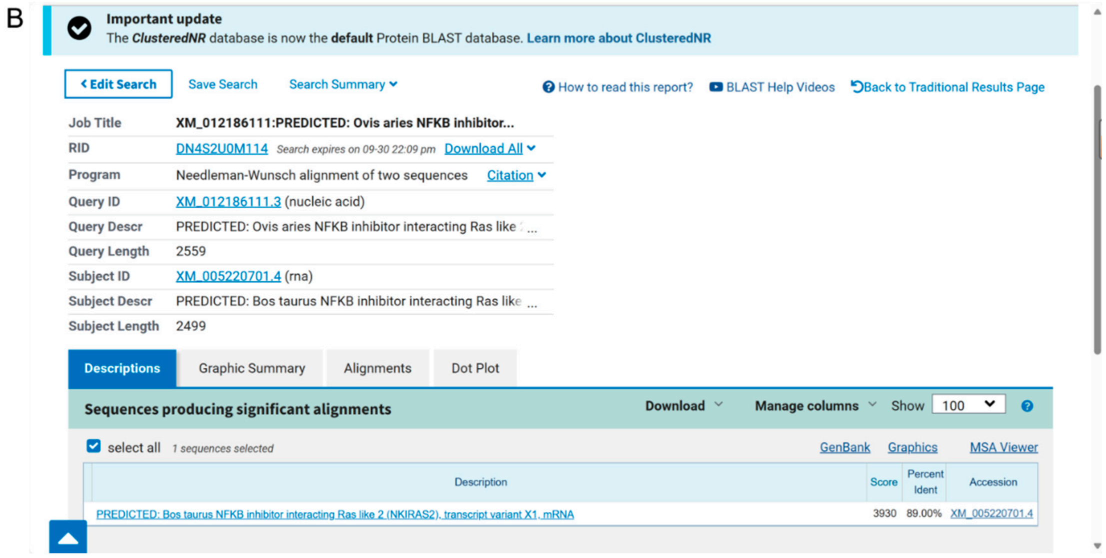

Figure S1. Homology comparison of oar-miR-125b and NKIRAS2 genes in sheep and cattle. (A) Alignment of mature sequences of oar-miR-125b in sheep and miR-125b in cattle. (B) The homology of NKIRAS2 mRNA in sheep and cattle.

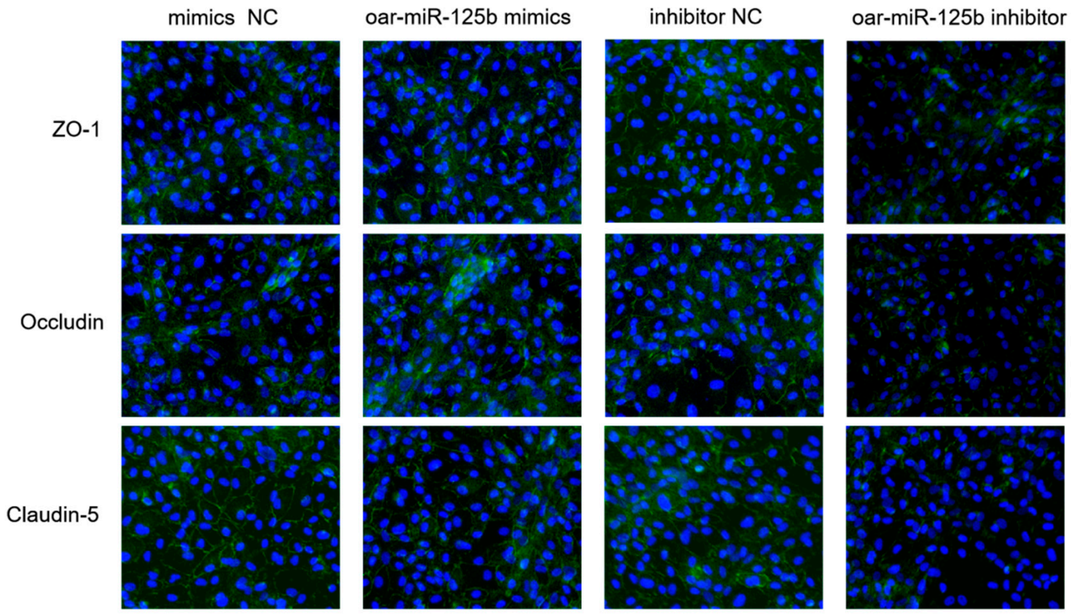

Figure S2. Oar-miR-125b regulates tight junction protein expression. Following transfection of OBMECs cells with oar-miR-125b mimics or inhibitors, immunofluorescence analysis was performed to detect the expression of tight junction-associated proteins.
